# Prevalence of Metabolic Syndrome Among Students: Associations with Dietary Habits, Physical Activity, and Sociodemographic Factors

**DOI:** 10.3390/jcm14134389

**Published:** 2025-06-20

**Authors:** Ema Dejhalla, Tina Zavidić, Branislava Popović, Tatjana Čulina

**Affiliations:** 1 Medical Centre for Occupational Health, Verdieva 8, 51000 Rijeka, Croatia; 2 Depatment of Family Medicine, Faculty of Medicine, University of Rijeka, B. Branchetta 20, 51000 Rijeka, Croatia; tina.zavidic@medri.uniri.hr (T.Z.); branislava.popovic@medri.uniri.hr (B.P.); tatjana.culina@medri.uniri.hr (T.Č.)

**Keywords:** metabolic syndrome, students, diet, physical activity

## Abstract

**Background/Objectives**: The prevalence of metabolic syndrome (MetS) among youth is rising, and the increase is closely linked to unhealthy lifestyle patterns. This study aimed to determine the prevalence of MetS among University of Rijeka students and investigate its associations with dietary habits, physical activity, gender, and faculty type (health and non-health faculties). **Methods**: A cross-sectional study conducted from September 2024 to March 2025 involved 217 randomly selected students from 16 faculties. The validated questionnaires Mediterranean Diet Adherence Screener (MEDAS) and International Physical Activity Questionnaire Short Form (IPAQ-SF), as well as a general data questionnaire, were used alongside anthropometric (height, weight, waist circumference) and biochemical measurements (fasting plasma glucose, triglycerides, HDL cholesterol). MetS was diagnosed using a combination of International Diabetes Federation (IDF) criteria and Polish Experts Consensus (2022) criteria. Statistical analyses included descriptive statistics, *t*-tests, ANOVA, Spearman’s correlation, and multivariate logistic regression. **Results**: MetS was identified in 5.5% of students. Significant risk factors included obesity (body mass index, BMI, *p* < 0.05), low physical activity (IPAQ-SF, *p* < 0.05), elevated blood pressure (*p* < 0.01), high triglyceride levels (*p* < 0.05), and increased waist-to-height ratio (WHtR, *p* < 0.01). Female students reported lower physical activity than males (*p* < 0.05), while students from non-health faculties had lower adherence to the Mediterranean diet (MEDAS, *p* < 0.05) and reduced physical activity (*p* < 0.05). Higher adherence to the Mediterranean diet correlated with lower BMI and triglyceride levels (*p* < 0.05), whereas lower adherence was associated with reduced physical activity (Spearman’s r = −0.35, *p* < 0.01). Logistic regression with WHR as the dependent variable showed waist circumference (WC) as the strongest predictor (OR = 45.925, 95% CI: 5.238–402.666, *p* = 0.001), followed by triglycerides (OR = 3.395, 95% CI: 1.322–8.718, *p* = 0.011). BMI was inversely associated with WHR (OR = 0.068, 95% CI: 0.006–0.780, *p* = 0.031). HDL cholesterol, systolic and diastolic blood pressure, and fasting glucose were not significant predictors (*p* > 0.05), indicating limited predictive power in this model. **Conclusions**: The 5.5% MetS prevalence underscores the need for targeted interventions promoting Mediterranean diet adherence and physical activity, particularly among non-health faculty students and females. Longitudinal studies are warranted to assess intervention efficacy.

## 1. Introduction

Metabolic syndrome (MetS) encompasses a combination of interrelated risk factors—including elevated blood pressure, central obesity, dyslipidemia, and impaired glucose metabolism—that collectively heighten the likelihood of developing cardiovascular disease and type 2 diabetes [1]. These factors represent an underlying disruption of metabolic processes, shaped by a complex interplay of genetic background, environmental influences, and lifestyle choices such as poor dietary habits and limited physical activity [2,3]. Although MetS is not a disease diagnosis per se, it is widely recognized as a strong early indicator of chronic disease risk and premature death.

Across Europe, MetS affects an estimated 13% to 36% of the population, with the highest rates observed in southern and eastern countries—likely linked to a higher obesity prevalence and unhealthy lifestyle trends in these regions. The prevalence of metabolic syndrome in Croatia varies depending on the study and criteria used, but it generally ranges from 34% to 42%. Studies on island populations have shown a prevalence of 34%, while a study in the Baranja region found a prevalence of 40% to 42% [4]. In young adults, prevalence rates range from 4.8% to 7% [5]. Although these numbers are lower than in older cohorts, the increasing incidence among youth is a growing public health concern, especially in the context of rising obesity and sedentary behavior [6]. This trend suggests that early-onset metabolic disturbances may lead to the earlier development of clinical complications, placing a significant burden on healthcare systems and reducing the quality of life in affected individuals [7].

MetS pathogenesis is multifactorial, with insulin resistance at its core—often driven by visceral adiposity. Excess abdominal fat acts as an endocrine organ, releasing pro-inflammatory cytokines and adipokines that impair glucose metabolism and vascular function. This creates a systemic pro-inflammatory, pro-atherogenic state that elevates long-term cardiovascular risk [7,8,9,10].

Lifestyle factors are strongly associated with the presence or absence of MetS. Among them, adherence to the Mediterranean diet, a dietary pattern rich in whole grains, fruits, vegetables, legumes, olive oil, and omega-3 fatty acids, has been consistently associated with a reduced MetS risk [11]. In addition, regular physical activity is associated with higher insulin sensitivity, improved lipid profiles, lower blood pressure, and reduced abdominal fat accumulation, as reported in prior studies [12]. These findings highlight the importance of health-promoting behaviors in mitigating cardiometabolic risk from an early age. Given the rising incidence of obesity and sedentary lifestyles among young adults, the early identification of MetS and its contributing factors is essential. Students represent a particularly relevant population, as this life stage is marked by increased autonomy in lifestyle choices, changes in dietary and physical activity habits, and the onset of lifelong health behaviors. Understanding the prevalence and determinants of MetS in this demographic is vital for designing effective public health interventions [13].

Various definitions of MetS have been proposed over time. The 1998 WHO criteria emphasized insulin resistance as a mandatory component, alongside at least two additional factors such as central obesity, dyslipidemia, hypertension, or microalbuminuria [14]. The NCEP ATP III (2001) definition simplified the diagnosis by requiring any three out of five specific components, without a mandatory one [15]. In contrast, the IDF criteria (2005) made central obesity essential (waist ≥ 94 cm for men, ≥80 cm for women), plus any two additional risk factors [16]. A 2009 joint statement by several international organizations harmonized the criteria by removing the requirement for abdominal obesity and allowing the diagnosis with any three out of five components, using region-specific waist cutoffs [17]. The most recent Polish criteria (2022) further adapted these definitions by including additional markers such as HbA1c and non-HDL cholesterol and adjusting blood pressure thresholds based on the measurement method [18].

This study applied a combination of the IDF (2005) and Polish (2022) definitions to reflect both international standards and regionally relevant clinical guidelines. This approach allowed for more accurate identification of MetS in the studied population, improving risk stratification and the applicability of findings in both local and broader public health contexts.

This study aimed to determine the prevalence of MetS among students at the University of Rijeka and evaluate the associations with dietary habits, particularly adherence to the Mediterranean diet, and levels of physical activity. Additionally, the study sought to examine differences by gender, faculty type (health and non-health faculties), and selected sociodemographic factors. Previous studies have not examined MetS prevalence among university students or differences by sex or faculty. International research in adolescents and young adults has consistently shown that poor diet and low physical activity increase MetS risk [19,20,21,22,23]. Studies from South Korea and Finland found that MetS prevalence rises with age and is linked to obesity and triglycerides, with stronger effects in women and younger adults [24,25]. A meta-analysis of 34 studies reported a 4.8–7% prevalence, with low HDL cholesterol as the most common component [26]. US data (1999–2018) has shown stable MetS rates but changing trends in its components [27].

This study is the first to assess how diet and physical activity influence MetS in university students, using validated questionnaires and analyzing differences by sex and faculty type.

Students from the University of Rijeka were selected due to a notable lack of existing research on metabolic syndrome within this population. While similar studies have been conducted among university students in other parts of Europe, epidemiological data on Croatian students—particularly in the Rijeka region—remain scarce. Addressing this gap provides context-specific insights that are crucial for developing locally adapted prevention strategies and public health interventions.

## 2. Materials and Methods

### 2.1. Study Design and Population

A cross-sectional study was conducted from September 2024 to March 2025, involving 217 students from 16 faculties at the University of Rijeka. The study was conducted in five family medicine practices and three school medicine practices in Croatia. Students who visited the selected family and school medicine practices were included in the study through random selection. The inclusion criteria were students at the University of Rijeka aged 18–24 years. The exclusion criteria were students who were immobile or had an active illness, fever, or treatment in progress.

The sample size was calculated using data indicating that the total number of students at the University of Rijeka is approximately 14,000, and the expected global prevalence of metabolic syndrome among young people ranges from 4.8% to 7%. With a 95% confidence interval, it was determined that a minimum of 100 students needed to participate in the study.

#### Ethical Considerations

The study was approved by the University of Rijeka Faculty of Medicine Ethics Committee, Number 007-08124-01/57, 2170-1-42-04-31-24-7, on 27 August 2024. Informed consent was obtained from all participants.

### 2.2. Data Collection

#### 2.2.1. Questionnaires

A general data questionnaire made by the authors was used to gather sociodemographic information, including age, sex, faculty, county of origin, presence of chronic diseases, medications, smoking status, diet location (cafeteria, restaurants, at home), and participation in professional sports.

The study utilized two more questionnaires to collect data. Adherence to the Mediterranean diet was assessed using the 14-item validated Mediterranean Diet Adherence Screener (MEDAS), with higher scores indicating greater adherence. The MEDAS questionnaire consists of 14 questions that assess various aspects of the Mediterranean diet, including the intake of specific foods and methods of food preparation. Each response is scored either a 0 or 1, depending on whether the habit aligns with the recommendations of the Mediterranean diet. The 14-item MEDAS questionnaire has been validated in multiple European countries, demonstrating consistent reliability and validity in measuring adherence to the Mediterranean diet. Studies comparing MEDAS with detailed dietary records and food frequency questionnaires have shown moderate to strong correlations (ICC typically 0.69–0.80), confirming its accuracy in reflecting actual dietary intake. Item-level agreement is particularly strong for key dietary components such as olive oil, fruits, and vegetables [28].

The consistent psychometric performance across diverse populations highlights MEDAS as a robust and adaptable tool for nutritional assessment. Its simplicity and efficiency make it especially valuable in large-scale epidemiological studies and public health interventions, where time and resources may be limited.

In summary, MEDAS is a scientifically validated, reliable, and practical instrument for evaluating Mediterranean diet adherence, supporting its widespread use in both clinical research and population-based health monitoring across Europe. A score of 9–14 points indicates high adherence to the Mediterranean diet, suggesting that the individual closely follows its core principles, such as a high intake of fruits, vegetables, legumes, whole grains, fish, and healthy fats like olive oil, with reduced consumption of red meat and processed foods. A score of 5–8 points indicates moderate adherence, meaning the individual partially follows the Mediterranean diet but could improve by increasing the intake of healthy foods. A low adherence score of 0–4 points suggests that the person does not significantly follow the Mediterranean dietary pattern [29,30,31,32,33].

Physical activity levels were assessed to examine their association with MetS risk factors, using the International Physical Activity Questionnaire Short Form (IPAQ-SF), which evaluates vigorous and moderate activity, walking, and sedentary time over the previous seven days and is validated for Croatian populations. The IPAQ collects information on the number of days per week and the amount of time spent daily on the following activities: vigorous physical activity (e.g., heavy lifting, aerobics), moderate physical activity (e.g., cycling at a moderate pace, carrying light loads), walking, and sitting. For each type of activity, the following parameters are recorded: frequency (number of days per week) and duration (minutes per day). Metabolic equivalent of task (MET) values are used to estimate energy expenditure for different activities: vigorous activity = 8.0 METs, moderate activity = 4.0 METs, and walking = 3.3 METs. High activity meets any of the following criteria: vigorous activity on at least 3 days accumulating at least 1500 MET-minutes/week, or 7 or more days of any combination of walking, moderate, or vigorous activity achieving a minimum of 3000 MET-minutes/week [34,35,36,37,38].

#### 2.2.2. Anthropometric Measurements and Medical Examination

Anthropometric measurements, including height, weight, and waist circumference, were obtained using International Society for the Advancement of Kinanthropometry (ISAK) protocols. From these measurements, several indices were calculated: Body mass index (BMI) was determined as weight in kilograms divided by height in meters squared. Body weight was measured using a validated calibrated digital floor scale, “Seca” (200 kg capacity, model 862), with an accuracy of 100 g. Body height was measured using a validated stadiometer, “Seca” (model 213). Waist circumference was measured to the nearest 0.1 cm using a cloth tailor’s measuring tape at the highest point of the iliac crest during minimal respiration, with the participant in a standing position. Hip circumference was measured at the point of the greatest buttock protrusion using the same type of measuring tape. The waist-to-hip ratio (WHR) was calculated as waist circumference divided by hip circumference, and the waist-to-height ratio (WHtR) was calculated as waist circumference divided by height, with a WHtR of ≥0.5 indicating central obesity [39]. Clinical measurements included blood pressure, which was assessed using the Omron M3 Comfort HEM-7134-E (Omron Healthcare Co., Kyoto, Japan).

#### 2.2.3. Laboratory Analysis

After a 12 h fast, a blood sample was taken from each participant through standard venipuncture performed by staff trained in blood collection. For the analysis of fasting plasma blood glucose concentration, triglycerides, and HDL cholesterol, 4 mL of venous blood was collected into vacuum tubes (BD Vacutainer^®^ Rapid Serum Tube (RST) Becton, Dickinson and Company, Franklin Lakes, NJ, USA) containing 1 mL of acid-citrate-dextrose (ACD) anticoagulant. The tubes were transported to the Teaching Institute of Public Health of the Primorje-Gorski Kotar County, where the samples were processed. Blood samples were centrifuged for at least 10 min at 3500× *g* at room temperature. For the analysis, Olympus AU2700 Plus and AU680 devices (Beckman Coulter, Tokyo, Japan), as well as Beckman Coulter analyzers (Beckman Coulter, Brea, CA, USA), were used. Fasting plasma glucose concentration was determined using an enzymatic UV test with the hexokinase method. Serum triglycerides and HDL cholesterol were analyzed using enzymatic colorimetric tests for quantitative determination (Roche Diagnostics GmbH, Mannheim, Germany).

### 2.3. MetS Diagnosis

MetS was diagnosed using a combination of International Diabetes Federation (IDF) criteria and Polish Experts Consensus criteria (2022) [27,28], as increasing migrations and population mixing in Europe necessitates a broader and more inclusive diagnostic framework [40,41].

The diagnosis required central obesity (waist circumference ≥ 102 cm for males and ≥88 cm for females, according to the Polish Experts Consensus criteria [2022]), plus at least two of the following components: blood pressure ≥ 130/85 mmHg (measured in clinic) or ≥130/80 mmHg (measured by patient); or hypertension treatment, fasting glucose ≥ 5.6 mmol/L; or glycemia after 2 h OGTT ≥ 140 mg/dL; or HbA1c ≥ 5.7%; or hypoglycemic treatment, HDL-C < 1.29 mmol/L (females) or <1.03 mmol/L (males); or hyperlipidemic therapy (according to the IDF criteria).

### 2.4. Statistical Analysis

Data analysis was performed using IBM SPSS Statistics, version 26.0. Before conducting comparisons, the distribution of numerical variables was assessed using a Shapiro–Wilk test to determine normality. Variables that followed a normal distribution were presented as mean ± standard deviation and analyzed using parametric tests (e.g., Student’s *t*-test, ANOVA). Variables that did not follow a normal distribution were described using the median and interquartile range (IQR) and analyzed with non-parametric tests (e.g., Mann–Whitney U test, Kruskal–Wallis test). Categorical variables were presented as frequencies and percentages and compared using a chi-square test or Fisher’s exact test where appropriate. Correlations between variables were assessed using Pearson’s or Spearman’s correlation coefficients, depending on the data distribution. A *p*-value of <0.05 was considered statistically significant.

## 3. Results

### 3.1. Participant Characteristics

**Figure 1:**
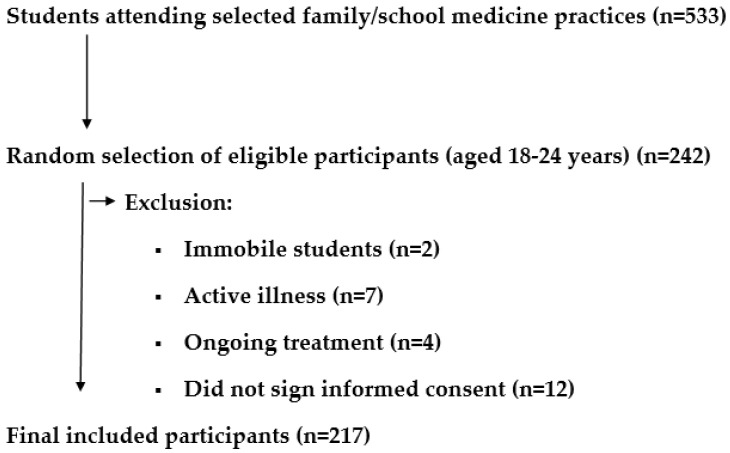
Participant recruitment.

Figure 1 shows the flowchart of participant recruitment.

Table 1 shows the age, gender, mean value of the observed variables, and distribution of participants according to BMI values.

Among the observed faculties, 50.2% of the participants attended a health-related faculty, while the remaining 49.8% were categorized as attending a non-health-related faculty. Health-related faculties include the Faculty of Medicine and the Faculty of Health Studies. Other faculties that were included in the study are the Academy of Applied Arts Rijeka, Faculty of Biotechnology and Drug Development, Faculty of Economics Rijeka, Faculty of Humanities and Social Sciences Rijeka, Faculty of Tourism and Hospitality Management, Faculty of Health Studies, Faculty of Civil Engineering Rijeka, Faculty of Medicine Rijeka, Faculty of Maritime Studies Rijeka, Faculty of Law Rijeka, Faculty of Engineering Rijeka, and Faculty of Teacher Education Rijeka. Considering the counties of origin, the highest proportion of participants came from Primorje-Gorski Kotar County (53.9%), followed by 9.2% from the state capital Zagreb and surrounding areas, and 9.2% from Istria County.

Regarding chronic illnesses, the most reported condition was hypothyroidism (seven participants). Furthermore, in terms of medications, the highest number of participants reported using levothyroxine (n = 7).

Additionally, 30.0% of the participants were smokers. Concerning meal locations, 56.2% reported eating at home, 41.9% in the cafeteria, and 1.8% in restaurants.

Table 2 presents the data for WC, HC, WHR, WHtR, systolic blood pressure, diastolic blood pressure, fasting plasma glucose, triglycerides, and HDL cholesterol. For each parameter, the arithmetic mean and standard deviation, minimum and maximum values, as well as the median with the corresponding interquartile range (IQR), are shown.

Furthermore, in terms of the Mediterranean diet, 28.6% of participants showed a low adherence, 58.1% had a moderate adherence, while 13.4% demonstrated a high adherence. Regarding physical activity, 13.4% had a low activity level, 41.2% had a moderate activity level, and 45.4% had a high activity level (Table 3).

### 3.2. Prevalence of Metabolic Syndrome

MetS prevalence was 5.5% (12/217 students), aligning with global estimates for young adults (4.8–7%) (Table 4) [2]. Prevalence was assessed using the IDF criteria, with recommendations for using waist circumference values of ≥102 cm in men and ≥88 cm in women.

When looking at the indicators for MetS, it can be observed that the highest proportion of positive findings was recorded for systolic blood pressure (34.6%), diastolic blood pressure (23.5%), triglycerides (14.3%), and HDL cholesterol (13.8%). Regarding systolic blood pressure, 41.5% of participants had normal values, 44.7% had elevated values, and 13.8% displayed hypertension. Similarly, for diastolic blood pressure, 15.7% had normal values, 72.4% had elevated values, and 12.0% presented hypertension. With respect to WHR, 62.2% of participants did not have elevated values, while 37.8% were at increased risk. For WHtR, 77.4% of participants had normal values, while 22.6% had elevated values (Table 5, Table 6 and Table 7).

#### Risk Factors and Statistical Associations

This study identified significant anthropometric, biochemical, and lifestyle risk factors associated with metabolic syndrome (MetS) among University of Rijeka students. Obesity, as indicated by a higher body mass index (BMI), was significantly associated with MetS (*p* < 0.05), showing a strong correlation with waist circumference (Spearman’s r = 0.783, *p* < 0.01; Table 8). Central obesity, defined by a waist-to-height ratio (WHtR) ≥ 0.5, emerged as a robust predictor of MetS (*p* < 0.01), with significant correlations to triglyceride level (r = 0.358, *p* < 0.01) and blood pressure (systolic: r = 0.357, *p* < 0.01; diastolic: r = 0.212, *p* < 0.01; Table 8). Elevated triglyceride levels (≥1.7 mmol/L) were significantly linked to MetS (*p* < 0.05), while increased blood pressure (systolic ≥ 130 mmHg or diastolic ≥ 85 mmHg) showed a strong association (*p* < 0.01). In contrast, low HDL cholesterol and elevated fasting glucose levels showed no significant associations with MetS (*p* > 0.05), suggesting a weaker role in this population.

A logistic regression analysis further quantified the strength of these associations, with waist-to-hip ratio (WHR) as the dependent variable, a key indicator related to MetS components. Waist circumference (WC) was a dominant predictor (OR = 45.925, 95% CI: 5.238–402.666, *p* = 0.001), indicating a substantial increase in the odds of an elevated WHR with a higher WC. Triglycerides were also significant (OR = 3.395, 95% CI: 1.322–8.718, *p* = 0.011), suggesting a more than threefold increase in the odds of an elevated WHR with elevated triglyceride levels. BMI was significantly associated with WHR (OR = 0.068, 95% CI: 0.006–0.780, *p* = 0.031), reinforcing its role in MetS risk. However, HDL cholesterol (OR = 2.511, 95% CI: 0.992–6.358, *p* = 0.052), systolic blood pressure (OR = 0.490, 95% CI: 0.227–1.058, *p* = 0.069), diastolic blood pressure (OR = 0.445, 95% CI: 0.177–1.118, *p* = 0.085), and fasting glucose (OR = 1.985, 95% CI: 0.449–8.771, *p* = 0.366) did not reach statistical significance at *p* < 0.05, indicating the limited predictive power for WHR in this model. The logistic regression model’s explanatory power is summarized in Table 9, with detailed results presented in Table 10.

Physical activity levels, assessed via the International Physical Activity Questionnaire Short Form (IPAQ-SF), were significantly associated with MetS (*p* < 0.05), with low activity levels increasing the odds of MetS prevalence. A significant negative correlation between adherence to the Mediterranean diet, measured by the Mediterranean Diet Adherence Screener (MEDAS), and physical inactivity (Spearman’s Q = −0.35, *p* < 0.01) underscores the interplay between lifestyle factors and MetS risk. These quantitative findings, supported by ORs and correlations, highlight BMI, central obesity (WHtR), elevated triglycerides, and low physical activity as key predictors of MetS, providing a robust foundation for subsequent analyses of sex and faculty differences in this study.

## 4. Discussion

This research marks the initial evaluation of the impact of dietary intake and physical activity on MetS among university students, employing validated questionnaires and examining variations across sex and faculty type. A distinctive feature of this study is that it represents the first such investigation conducted in Croatia.

### 4.1. Prevalence of Metabolic Syndrome and Related Health Metrics

This study found a MetS prevalence of 5.5% among 217 students at the University of Rijeka, aligning with prior research reporting 4.7% to 7% prevalence in young adults [2]. Among those meeting MetS criteria (including mandatory abdominal obesity), the most common components were elevated systolic (34.6%) and diastolic (23.5%) blood pressure, triglycerides (14.3%), and reduced HDL-cholesterol (13.8%). Unlike findings by Sapkota M. et al. and Li C. et al., where reduced HDL was the dominant criterion [42,43], this study showed blood pressure abnormalities as the most prominent. Only 41.5% of participants had normal systolic blood pressure, and only 15.7% had normal diastolic values, suggesting early dysregulation potentially tied to sedentary behavior, stress, dietary habits, and stimulant use. Over 70% of students had elevated diastolic pressure, and over 10% met hypertension criteria, indicating a pressing need for preventive strategies including education, physical activity promotion, and improved dietary habits. WHR indicated increased risk in 37.8% of students, while 22.6% had elevated WHtR, both markers of cardiometabolic vulnerability.

### 4.2. Lifestyle Factors: Diet, Physical Activity, and Smoking

Lifestyle factors were significantly associated with MetS risk. Mediterranean diet adherence was low in 28.6%, moderate in 58.1%, and high in only 13.4%, reflecting challenges such as limited time, budgets, and reliance on fast food or cafeterias. High adherence, though limited, indicates some awareness of healthy eating. Physical activity levels were low in 13.4%, moderate in 41.2%, and high in 45.4%, with students with MetS being less active, consistent with previous studies [44]. Meal location further reflected habits: 56.2% ate at home, 41.9% in cafeterias, and only 1.8% in restaurants. Students from Primorje-Gorski Kotar County often ate at home, suggesting better nutritional control. Smoking was reported by 30.0% of participants and was associated with increased waist circumference and triglyceride levels, confirming its adverse effects on metabolic health.

### 4.3. Gender and Faculty Differences

The sample included more women (63.6%), who were significantly less physically active (75.9%, *p* < 0.05), aligning with Peng B. et al. [45]. Men had higher BMI and obesity prevalence. Students from non-health faculties had lower Mediterranean diet adherence (64.5%, *p* < 0.05), less physical activity (48.3%, *p* < 0.05), and higher central adiposity indicators, while health faculty students showed higher diastolic blood pressure and HDL-cholesterol, possibly due to greater health awareness. Chi-square analysis showed significant differences (*p* < 0.05) in BMI, physical activity, blood pressure, and WHtR related to MetS presence. Regression analyses highlighted waist circumference, triglycerides, and BMI as predictors of WHR, while triglycerides, systolic blood pressure, and BMI predicted WHtR. Strong correlations were observed between waist circumference and HDL-cholesterol (r = −0.455), WHtR and HDL-cholesterol (r = −0.387), and waist circumference and WHtR (r = 0.929), underscoring the impact of abdominal obesity.

### 4.4. Comparisons with Prior Literature

Our findings are broadly consistent with international research. Studies from the Basque region, Poland, and southeast Europe report similar or slightly lower obesity and overweight prevalence among students [44,45,46,47]. This study’s physical activity results (13.4% low, 41.2% moderate, 45.4% high) are comparable to those reported by Padmapriya K. et al. [44]. Mediterranean diet adherence findings mirror those by Antonopoulou et al. [46]. Meal location preferences are also similar to those from Yun T.C.’s study in Brunei [48]. The 30.0% smoking rate and presence of chronic conditions like hypothyroidism further contextualize health risks. Geographic distribution, with over half the sample from Primorje-Gorski Kotar County, reflects university proximity and economic factors. These patterns suggest that students in Rijeka follow regional and global trends in health behavior, though high blood pressure prevalence and lower HDL levels may reflect local lifestyle influences.

### 4.5. Limitations and Future Directions

The study’s cross-sectional design limits causal inference, and reliance on self-reported questionnaires introduces possible recall bias. Though the sample is representative, broader generalizability is limited. Future research should use longitudinal designs and larger samples and investigate genetic and psychosocial influences on MetS development. Preventive programs promoting diet, physical activity, and other healthy behaviors are urgently needed to mitigate MetS risk among university students.

## 5. Conclusions

MetS affects 5.5% of University of Rijeka students and is associated with obesity (BMI, *p* < 0.05), low physical activity (*p* < 0.05), high blood pressure (*p* < 0.01), elevated triglyceride levels (*p* < 0.05), and WHtR (*p* < 0.01). Females and non-health faculty students exhibit less favorable lifestyle patterns, indicating the need for tailored health promotion efforts. While causality cannot be inferred due to the cross-sectional design, the findings suggest that Mediterranean diet adherence and regular physical activity are associated with characteristics that may relate to lower MetS prevalence. University programs could incorporate integrated wellness initiatives—such as nutrition education, accessible physical activity opportunities, and targeted outreach to at-risk groups—to help reduce MetS risk. Longitudinal research is recommended to confirm these associations and inform evidence-based public health strategies.

## Figures and Tables

**Table 1:** Age, gender, mean value of the observed variables, and distribution of participants according to BMI values.

Variable							
Age (years)	Mean: 21.43	SD: 2.07	Min: 18	Max: 24	25th: 19.00	Median: 21.00	75th: 24.00
Gender	Male: 79 (36.4%)	Female: 138 (63.6%)	Total: 217 (100%)				
Height (cm)	Mean: 173.68	SD: 10.12	Min: 154.00	Max: 200.00	25th: 165.00	Median: 173.00	75th: 182.00
Weight (kg)	Mean: 72.74	SD: 14.73	Min: 47.00	Max: 116.00	25th: 60.00	Median: 70.00	75th: 85.00
BMI (kg/m^2^)	Mean: 23.95	SD: 3.42	Min: 16.19	Max: 37.45	25th: 21.65	Median: 23.72	75th: 25.96
BMI Categories	Underweight: 9 (4.1%)	Normal: 152 (70.0%)	Overweight: 26 (12.0%)	Obesity I: 27 (12.4%)	Obesity II: 3 (1.4%)		

Abbreviations: SD—standard deviation; Min—minimum; Max—Maximum; BMI—body mass index.

**Table 2:** Data for the indicators of metabolic syndrome.

	$\bar{x}$	Sd	Min	Max	Percentiles		
					25	50	75
WC (cm)	78.66	12.972	57	126	68.50	77.00	87.00
HC (cm)	93.90	11.886	72	131	85.00	93.00	102.00
WHR	0.8372	0.08130	0.65	0.98	0.7700	0.8300	0.9100
WHtR	0.4517	0.06287	0.34	0.75	0.4050	0.4500	0.4900
systolic blood pressure (mmHg)	123.2673	14.38597	95.00	171.00	113.0000	120.0000	133.0000
diastolic blood pressure (mmHg)	76.9401	9.13688	59.00	110.00	70.0000	76.0000	84.0000
fasting glucose (mmol/L)	4.6848	0.49684	2.80	6.30	4.4000	4.6000	5.0000
triglycerides (mmol/L)	1.0795	0.66392	0.20	4.80	0.7000	0.9000	1.3000
HDL cholesterol (mmol/L)	1.4977	0.33131	0.50	2.60	1.3000	1.5000	1.7000

Abbreviations: WC—waist circumference; HC—hip circumference; WHR—waist-to-hip ratio; WHtR—waist-to-height ratio; HDL—high-density lipoprotein.

**Table 3:** Distribution of participants according to diet and physical activity.

		N	%
diet	low adherence	62	28.6%
	moderate adherence	126	58.1%
	high adherence	29	13.4%
	Total	217	100.0%
physical activity	low activity	29	13.4%
	moderate activity	89	41.2%
	high activity	98	45.4%
	Total	216	100.0%

Abbreviation: N—number.

**Table 4:** Prevalence of metabolic syndrome.

		N	%
metabolic syndrome	yes	12	5.5%
	no	205	94.5%
	Total	217	100.0%

Abbreviations: N—number.

**Table 5:** Indicators of metabolic syndrome.

		N	%
WC (cm)	no	199	91.7%
	yes	18	8.3%
	Total	217	100.0%
fasting glucose (mmol/L)	no	206	94.9%
	yes	11	5.1%
	Total	217	100.0%
triglycerides (mmol/L)	no	186	85.7%
	yes	31	14.3%
	Total	217	100.0%
HDL-cholesterol (mmol/L)	no	187	86.2%
	yes	30	13.8%
	Total	217	100.0%
systolic blood pressure (mmHg)	no	142	65.4%
	yes	75	34.6%
	Total	217	100.0%
diastolic blood pressure (mmHg)	no	166	76.5%
	yes	51	23.5%
	Total	217	100.0%
BMI (m/kg^2^)	<30	204	94.0%
	≥30	13	6.0%
	Total	217	100.0%

Abbreviations: WC—waist circumference; HDL—high-density lipoprotein; BMI—body mass index.

**Table 6:** Systolic and diastolic pressure.

		N	%
systolic blood pressure (mmHg)	normal values	90	41.5%
	elevated values	97	44.7%
	hypertension	30	13.8%
	Total	217	100.0%
diastolic blood pressure (mmHg)	normal values	34	15.7%
	elevated values	157	72.4%
	hypertension	26	12.0%
	Total	217	100.0%

**Table 7:** Data for WHR and WHtR.

		N	%
WHR	normal values	135	62.2%
	increased risk	82	37.8%
	Total	217	100.0%
WHtR	normal values	168	77.4%
	elevated values	49	22.6%
	Total	217	100.0%

Abbreviations: WC—waist circumference; WHR—waist-to-hip ratio.

**Table 8:** Spearman’s correlation coefficients between the observed variables.

		WC (cm)	HC (cm)	WHR	WHtR	Systolic Blood Pressure (mmHg)	Diastolic Blood Pressure (mmHg)	Fasting Glucose (mmol/L)	Triglycerides (mmol/L)	HDL-Cholesterol (mmol/L)
1. WC (cm)	r	1.000	0.783 **	0.606 **	0.929 **	0.425 **	0.251 **	0.200 **	0.357 **	−0.455 **
	*p*	.	0.000	0.000	0.000	0.000	0.000	0.003	0.000	0.000
	N	217	217	217	217	217	217	217	217	217
2. HC (cm)	r	0.783 **	1.000	0.008	0.664 **	0.521 **	0.379 **	0.155 *	0.254 **	−0.340 **
	*p*	0.000	.	0.905	0.000	0.000	0.000	0.022	0.000	0.000
	N	217	217	217	217	217	217	217	217	217
3. WHR	r	0.606 **	0.008	1.000	0.645 **	0.059	−0.030	0.147 *	0.274 **	−0.304 **
	*p*	0.000	0.905	.	0.000	0.385	0.661	0.031	0.000	0.000
	N	217	217	217	217	217	217	217	217	217
4. WHtR	r	0.929 **	0.664 **	0.645 **	1.000	0.357 **	0.212 **	0.195 **	0.358 **	−0.387 **
	*p*	0.0000	0.000	0.000	.	0.000	0.002	0.004	0.000	0.000
	N	217	217	217	217	217	217	217	217	217
5. Systolic blood pressure (mmHg)	r	0.425 **	0.521 **	0.059	0.357 **	1.000	0.637 **	0.130	0.229 **	−0.195 **
	*p*	0.000	0.000	0.385	0.000	.	0.000	0.056	0.001	0.004
	N	217	217	217	217	217	217	217	217	217
6. Diastolic blood pressure (mmHg)	r	0.251 **	0.379 **	−0.030	0.212 **	0.637 **	1.000	0.051	0.226 **	−0.207 **
	*p*	0.000	0.000	0.661	0.002	0.000	.	0.451	0.001	0.002
	N	217	217	217	217	217	217	217	217	217
7. Fasting glucose (mmol/L)	r	0.200 **	0.155 *	0.147 *	0.195 **	0.130	0.051	1.000	0.202 **	−0.072
	*p*	0.003	0.022	0.031	0.004	0.056	0.451	.	0.003	0.290
	N	217	217	217	217	217	217	217	217	217
8. Triglycerides (mmol/L)	r	0.357 **	0.254 **	0.274 **	0.358 **	0.229 **	0.226 **	0.202 **	1.000	−0.322 **
	*p*	0.000	0.000	0.000	0.000	0.001	0.001	0.003	.	0.000
	N	217	217	217	217	217	217	217	217	217
9. HDL-cholesterol (mmol/L)	r	−0.455 **	−0.340 **	−0.304 **	−0.387 **	−0.195 **	−0.207 **	−0.072	−0.322 **	1.000
	*p*	0.000	0.000	0.000	0.000	0.004	0.002	0.290	0.000	.
	N	217	217	217	217	217	217	217	217	217

Abbreviations: WC—waist circumference; HC—hip circumference; WHR—waist-to-hip ratio; WHtR—waist-to-height ratio; HDL—high-density lipoprotein. ** Correlation is significant at the 0.01 level (2-tailed). * Correlation is significant at the 0.05 level (2-tailed).

**Table 9:** Model summary (dependent variable WHR).

Step	−2 Log Likelihood	Cox and Snell R Square	Nagelkerke R Square
1	243.426 ^a^	0.185	0.252

Abbreviations: WHR—waist-to-hip ratio. ^a^ Estimation terminated at iteration number 6 because parameter estimates changed by less than 0.001.

**Table 10:** Logistic regression analysis of factors associated with waist-to-hip ratio (WHR) as the dependent variable.

Variable	B	S.E.	Wald	df	Sig.	Exp(B)	95% CI for Exp(B)
WC	3.827	1.108	11.936	1	0.001	45.925	5.238–402.666
Fasting Glucose	0.686	0.758	0.818	1	0.366	1.985	0.449–8.771
Triglycerides	1.222	0.481	6.451	1	0.011	3.395	1.322–8.718
HDL-Cholesterol	0.921	0.474	3.773	1	0.052	2.511	0.992–6.358
Systolic Blood Pressure	−0.713	0.392	3.302	1	0.069	0.490	0.227–1.058
Diastolic Blood Pressure	−0.809	0.470	2.967	1	0.085	0.445	0.177–1.118
BMI	−2.689	1.245	4.663	1	0.031	0.068	0.006–0.780
Constant	−0.606	0.198	9.347	1	0.002	0.545	–

Exp(B) represents the odds ratio (OR) associated with each variable. A 95% confidence interval (CI) that crosses 1.0 suggests a lack of statistical significance at the *p* < 0.05 level. Model fit: Nagelkerke R^2^ = 0.252, indicating an adequate fit. The logistic regression model for WHR showed a good fit (Nagelkerke R^2^ = 0.32), with BMI as a significant predictor (*p* = 0.031). Variable(s) entered: WC—Waist circumference, Fasting glucose, Triglycerides, HDL-cholesterol (HDL—high-density lipoprotein), Systolic blood pressure, Diastolic blood pressure, BMI—Body mass index.

## Data Availability

Further information concerning the present study is available from the corresponding authors upon reasonable formal request.
